# Effects of 1,8-cineole on Carbohydrate Metabolism Related Cell Structure Changes of *Salmonella*

**DOI:** 10.3389/fmicb.2018.01078

**Published:** 2018-05-25

**Authors:** Yangying Sun, Xiaojun Cai, Jinxuan Cao, Zhen Wu, Daodong Pan

**Affiliations:** ^1^Key Laboratory of Animal Protein Deep Processing Technology of Zhejiang Province, Ningbo University, Zhejiang, China; ^2^Department of Food Science and Nutrition, Jinling College, Nanjing Normal University, Nanjing, China

**Keywords:** 1,8-cineole, carbohydrate metabolism, cell structure, *Salmonella*, proteomics

## Abstract

*Salmonella* is gram-negative foodborne zoonotic bacteria which triggers disease in humans. Our previous studies showed 1,8-cineole possessed remarkable antimicrobial effects on foodborne zoonotic bacteria indicating it could serve as a new source of antibiotic for *Salmonella*. Present study elucidated the antibacterial mechanism of 1,8-cineole by analyzing serum protein expressed by *Salmonella* following treatment with 1,8-cineole (0.25 mg/mL, 3 h) using isobaric tags for relative and absolute quantification (iTRAQ) with two-dimensional liquid chromatography/tandem mass spectrometry (2D-LC-MS/MS). 1,8-cineole was found to significantly damage the structure of *Salmonella* cell walls and membranes. A total of 3011 proteins were extracted from the experimental group, of which 435 were differentially expressed (1.5-fold) with 123 upregulated and 312 downregulated. The expressed proteins were involved in 935 intracellular biological processes, 98 cellular components, 477 molecular functions and 86 Kyoto Encyclopedia of Genes and Genomes (KEGG) pathways. Among them, proteins associated with carbohydrate, nucleotide, amino acid, lipid, and energy metabolism were significantly changed following treatment with 1,8-cineole. Carbohydrate metabolism and membrane protein-related genes was down-regulated at the mRNA level when *Salmonella* was treated with 1,8-cineole. 1,8-cineole may be a potential antibiotic for *Salmonella* infections.

## Introduction

*Salmonella* is a common gram-negative foodborne zoonotic bacterium with more than 2,500 serotypes (Eng et al., 2015). It mainly lives in animal intestine and easily contaminates animal products (poultry meat, beef, pork, eggs, and milk), fresh fruits, vegetables, nuts, and grains. Raw meat is sensitive to *Salmonella* infection (Chai et al., 2011; Silva et al., 2011; Nsoesie et al., 2014). Humans are infected with *Salmonella* primarily through contact or by eating contaminated fresh meat products (Silva et al., 2011; Antunes et al., 2016). *Salmonella* infections may trigger acute inflammatory response (Santos, 2014), enteric fever, enterogastritis, bacteremia, parenteral complications, or may lead to chronic carrier state (Eng et al., 2015). Severity of the reaction mainly depends on the individual's health status and immune ability. *Salmonella* Typhi, *Salmonella* Paratyphi, *Salmonella* Typhimurium, and *Salmonella* Enteritidis can cause life-threatening diseases in certain populations, including infants, seniors, and patients with compromised immune systems (Chen et al., 2013; Crump et al., 2015). However, owing to overuse of antibiotics and increasing microbial resistance to antibiotics, new antibiotics against microbial infections are urgently needed (Gould and Bal, 2013).

New proteomic techniques, such as isobaric tags for relative and absolute quantification (iTRAQ), liquid chromatography tandem mass spectrometry (LC-MS/MS), and matrix-assisted laser desorption/ionization time-of-flight (MALDI-TOF), have paved new pathways toward discovering new drugs for microbial-induced infectious diseases (Chen et al., 2017).

Due to their antimicrobial activity against foodborne pathogens, essential oils (EOs), and their components have attracted a great deal of attention (Burt, 2004; Ojeda-Sana et al., 2013; Bassanetti et al., 2017; Hac-Wydro et al., 2017). These compounds have been used as natural food preservatives in recent years. Antibacterial mechanism and target sites of terpenoids in EOs mainly focus on bacterial cell membrane, cytoplasm, and cell morphology by affecting fatty acids in the cell membrane, as well as proteins, ATP and ATPases, metabolome, cell morphology, and anti-quorum sensing activity (Nazzaro et al., 2013). 1,8-cineole (CIN), also known as eucalyptol or cajeputol, is a flavoring agent and antimicrobial monoterpene compound found in many natural-plant EOs (Mulyaningsih et al., 2010; Teixeira et al., 2013). CIN showed significant antimicrobial activity against human pathogens including *Bacillus subtilis, Enterobacter cloacae, Escherichia coli* O157:H7, *Proteus mirabilis, Pseudomonas aeruginosa*, and *Salmonella typhimurium*, by disrupting the cell membrane's permeability (Soković et al., 2010; Ojeda-Sana et al., 2013).

Present research aimed to determine the effects of *Salmonella* exposure to the essential components of CIN oil. We analyzed the carbohydrate metabolic pathways and antibacterial mechanisms of CIN against *Salmonella*-induced diseases by comparing the differences in terms of differential protein expression levels of treated and untreated bacteria. Understanding of *Salmonella* proteomics in relation to CIN exposure will lead to further research and development of new antibacterial agents.

## Materials and methods

### Bacterial strain and growth conditions

*Salmonella* bacteria (*Salmonella* sp. D194-2) were purchased from the China General Microbiological Culture Collection Center. The bacteria were cultured according to the procedures previously described by Reddy et al. (2015). Briefly, *Salmonella* cultures were transferred into 2–100 mL fresh sterile NB broth and incubated to OD_600_ = 0.4 after culturing in NB broth medium (Hope Bio-Technology, Qingdao, China) at 37°C. The bacteria of 0.4 OD were 10^6^–10^7^ CFU/mL. After overnight culture, *Salmonella* sp. D194-2 were inoculated to 75 mL sterile broth with 2% inoculation. Following that, they were incubated in a thermostatic oscillation incubator for 4–5 h at a speed of 150 r/min at 37°C to reach 0.4 ± 0.01 OD at 600 nm. The concentration of bacteria suspension were 10^6^–10^7^ CFU/mL in our experiments. CIN (99%, J&K Scientific Ltd., Beijing, China) was dissolved in 10% ethanol-broth solution to prepare a mother solution with a concentration of 10 mg/mL and then diluted to 1×MIC (2.5 mg/mL CIN, 2.5% ethanol). MICs were determined using a micro-broth dilution assay in a previous study. The control group 0 × MIC was prepared with 2.5% ethanol-broth solution, which was four times dilution with 10% ethanol-broth solution. The compound at concentrations of 0 and 2.5 mg/mL (0×MIC and 1×MIC, respectively) were added to the bacterial cultures and incubated. The incubation temperature was 37°C. Control group (0×MIC) incubated at 0 h and experimental group (1×MIC) incubated at 3 and 6 h were used for transmission electron microscope (TEM) analysis. Meanwhile, both control and experimental groups (0×MIC and 1×MIC) incubated at 3 h were used for proteomic sample preparation. The bacteria suspensions were centrifuged (Xiang Yi H-2050R, Hunan, China) at 5,000 r/min for 10 min at 4°C for transmission electron microscopic (TEM) observation and total protein analysis. The experiments were performed in triplicate.

### Transmission electron microscope (TEM) analysis

Harvested bacteria (from 100 mL culture medium) from 1 × MIC groups were incubated in a thermostatic oscillation incubator which was shake at a speed of 150 r/min at 37°C for 3 and 6 h, Meanwhile, control group (0×MIC) was incubated at 0 h. Bacteria suspensions were centrifuged at 5,000 r/min for 10 min at 4°C, and the resulting precipitation was washed three times with 0.1 M phosphate buffer saline (PBS, pH 7.4, Solarbio, China). Samples were fixed in 2.5% (v/v) glutaraldehyde at 4°C for 2 h. After rinsing in 0.1 M phosphate buffer saline (pH 7.4) for four times (15 min/step), samples were fixed with 1% (w/v) osmic acid at 4°C in the dark for 1 h. Samples were then centrifuged to discard the suspension and rinsed with PBS three times. Finally, samples were dehydrated in a graded ethanol series [15 min/step; (30–50–70–80–90)% (v/v)], acetone solution [90% (v/v)], and then dehydrated three times with anhydrous acetone (15 min/step). After embedding, drying, slicing, metal spraying and coloring, samples were observed with a TEM (Hitachi JEM-1230, Japan) using an accelerating voltage of 200 kV.

### Proteomic sample preparation

Preparations of proteomic sample were carried out at 4°C. *Salmonella* sp. D194-2 bacteria from the control (0×MIC) and the experimental (1× MIC) groups were incubated at 37°C for 3 h and harvested by centrifuging at 5,000 r/min for 15 min and then washed with PBS (0.1M, pH 7.4) three times before total protein extraction. Sample pellets (0.1 g) were lysis in solution containing 8 M urea, 1% dithiothreitol (DTT) and a protease inhibitor (Sigma, USA). After ultra-sonication on ice for 3 min (80 W, on 0.8 s/off 0.8 s), 1 mL sample lysis was well-distributed into 5 mL cooled acetone at −20°C for 2 h and centrifuged at 12,000 × g for 10 min at 4°C. The collected precipitates were fixed with cooled acetone and centrifuged at 12,000 × g for 15 min at 4°C, and then this step was repeated. Total protein was extracted by lysing dry precipitates in lysis solution and centrifuged twice (12,000 × g, 15 min). The supernatant liquid was the extracted protein solution. Concentrations of the protein extracts were determined using BCA method with a Bradford Protein Assay Kit (Sangon Biotech, Shanghai, China) (Smith et al., 1985).

### iTRAQ labeling and fractionation

iTRAQ labeling and fractionation were conducted using the method previously described by Wiśniewski et al. (2009). Protein samples were firstly digested at 37°C with Trypsin Gold (Promega, Madison, WI) for 12 h (50 ng/μL). Each protein sample (100 μg) were dissolved in cooled acetone (1:5 volume ratio) at −20°C for 1 h and centrifuged (12,000 × g, 10 min, 4°C) and freeze dried under vacuum condition. After reductive alkylation and enzymolysis, peptides were processed and obtained according to iTRAQ 8-plex kit (AB SCIEX, USA). All labeling peptides mixed with iTRAQ reagent were swirled, centrifuged and dried in a freeze dryer (Thermo Savant, USA). Freeze-dried mixtures were dissolved into 110 μL acetonitrile (ACN) solution (Thermo Fisher Scientific, USA) and fractionated using an Agilent 1200 High Performance Liquid Chromatography (HPLC) System (Agilent, USA). The eluted peptides were separated into 10 segments which were then dried in a vacuum freeze dryer.

### Nanobore reversed-phase liquid chromatography tandem mass spectrometry (nano-RPLC-MSMS) analysis and protein identification 2D-LC-MSMS

Online Nano-RPLC was used on the Eksigent nanoLC-Ultra™ 2D System (AB SCIEX, USA). Freeze-dried peptides re-suspended with Nano-RPLC buffer A (2% ACN, 0.1% FA) were loaded onto a C18 nanoLC trap column (100μm × 3cm, C18, 3 μm, 150 Å) at a flow rate of 2 μL/min and de-salted for 10 min at the flow rate. The elution gradient was as follows: 0 min: 5% B (98%ACN, 0.1% FA), 70 min: 35%B. ChromXP C18 column (75μm × 15cm, C18, 3μm 120 Å) (ChromXP Eksigent, AB SCIEX, USA) with a spray tip was used for separation of the peptides. Data acquisition was performed with Triple TOF 5600 System (AB SCIEX, USA) fitted with a Nanospray III source (AB SCIEX, USA) and a pulled quartz tip as the emitter (New Objectives, USA). The data was acquired using an ion spray voltage of 2.5 kV, curtain gas of 30 PSI, nebulizer gas of 5 PSI, and an interface heater temperature of 150°C. For information dependent acquisition (IDA), survey scans were acquired at 250 MS and as many as 35 product ion scans were collected if they exceeded a threshold of 150 counts per second (counts/s) with a 2^+^ to 5^+^ charge-state. The total cycle time was fixed to 2.5 s. A rolling collision energy setting was applied to all precursor ions for collision-induced dissociation (CID). Dynamic exclusion was set for half of the peak width (18 s). The precursor was then refreshed off the exclusion list.

The experimental data from tandem mass spectrometry (MS) (wiff files) was processed with Protein Pilot Software v. 5.0 (AB SCIEX, USA) against a *Salmonella* database using Paragon algorithm for protein and peptides identification (Shilov et al., 2007). Data with at least 95% confidence of identification was applied for identification of unique peptides and proteins. The proteins identified were screened and classified according to UniProt online database (http://www.uniprot.org/). Functional classification and analysis of differential proteins identified were based on Gene Ontology (GO) Terms (http://www.geneontology.org/). Systematic interpretation of differentially expressed proteins was analyzed using the Kyoto Encyclopedia of Genes and Genomes (KEGG) (http://www.genome.jp/kegg/pathway.html) and mapped for Protein-Protein Interaction (PPI) using the String Database (http://string.embl.de/).

### Validation of proteomics analysis

Total RNA was extracted using a Bacterial RNA Kit (OMEGA Biotek, USA). The reverse transcription of the RNA was performed with a TransScript All-in-One First-Strand cDNA Synthesis Super Mix kit (Rayscript cDNA Synthesis KIT, Shanghai Generay Biotech Co., Ltd, China). The qPCR was implemented in a TransStart Tip Green qPCR SuperMix kit (TransGen Biotech, China) and quantified with a LightCycler 96 (Roche, Switzerland). The quantitative PCR cycle threshold (CT) results were analyzed using the comparative CT method (2^−ΔΔCT^ method). All kits were used according to the manufacturers' instructions. Primers used for RT-PCR are listed in Table S4.

### Statistics

Functional classifications of the proteins were analyzed using bioinformatic method. The proteins identified were screened and classified according to the UniProt online database (http://www.uniprot.org/). Functional classification and analysis of differential proteins identified were based on Gene Ontology (GO) Terms (http://www.geneontology.org/). The Kyoto Encyclopedia of Genes and Genomes database (KEGG, http://www.genome.jp/kegg/pathway.html) were applied for pathway annotation. Protein-protein interaction (PPI) network was illustrated by String Database (http://string.embl.de/). The differential proteins identified were screened and classified according to online database David 6.7 (http://david.abcc.ncifcrf.gov/) and QuickGO (http://www.ebi.ac.uk/QuickGO/). Differentially expressed proteins were mapped to KEGG database (http://www.kegg.jp/kegg/pathway.html). According to the three metabolism pathways, Glycolysis/Gluconeogenesis (eco00010), Pyruvate metabolism (eco00620), and Citrate cycle (TCA cycle) (eco00020), in KEGG database, carbohydrate metabolism figure was obtained in the KEGG pathways with up-regulated and down-regulated proteins. Differentially expressed proteins with at or more than 1.5-fold were considered significantly up-regulated, meanwhile proteins differentially expressed at or less than 0.67-fold were considered to be significantly down-regulated. Origin8.5 (OriginLab, USA), Microsoft office PowerPoint 2007 and Microsoft Excel 2007 were employed for data processing. Proteins enriched by OmicsBean (http://www.omicsbean.cn, Gene for Health Ltd., Shanghai, China) with computed *p*-values less than 0.05 were considered as significant in GO terms and KEGG pathway. Enrichment Analysis of GO terms and KEGG pathway were conducted by the hypergeometric test with OmicsBean. For example, an Enrichment Analysis is to test whether a GO term is statistically enriched for the given set of genes, the hypergeometric test is the most common statistic method of enrichment analysis. Where N is the number of all genes of the specific organism that were annoted in GO (Background Genes); n is the number of query genes annotated to the GO Term; M is the number of all genes that are annotated to certain GO terms (Pop Hit); m is the number of query genes annoted to certain GO terms (count). The formula was as follows: $P=1-\overset{m-1}{\underset{i=0}{\sum \text{​}}}\frac{\left(\begin{array}{c} M\\ i \end{array}\right)\left(\begin{array}{c} N-M\\ n-i \end{array}\right)}{\left(\begin{array}{c} N\\ n \end{array}\right)}$. Benjamini-Hochberg Method for Multiple Hypothesis Testing was also applied in Enrichment Analysis of GO terms and KEGG pathway to adjust *p*-values. Protein expression levels were analyzed by two-sample *T*-test with Protein Pilot 5.0 (AB SCIEX, USA).

## Results

### Effects on the morphology of bacterial cells

**Figure 1:**
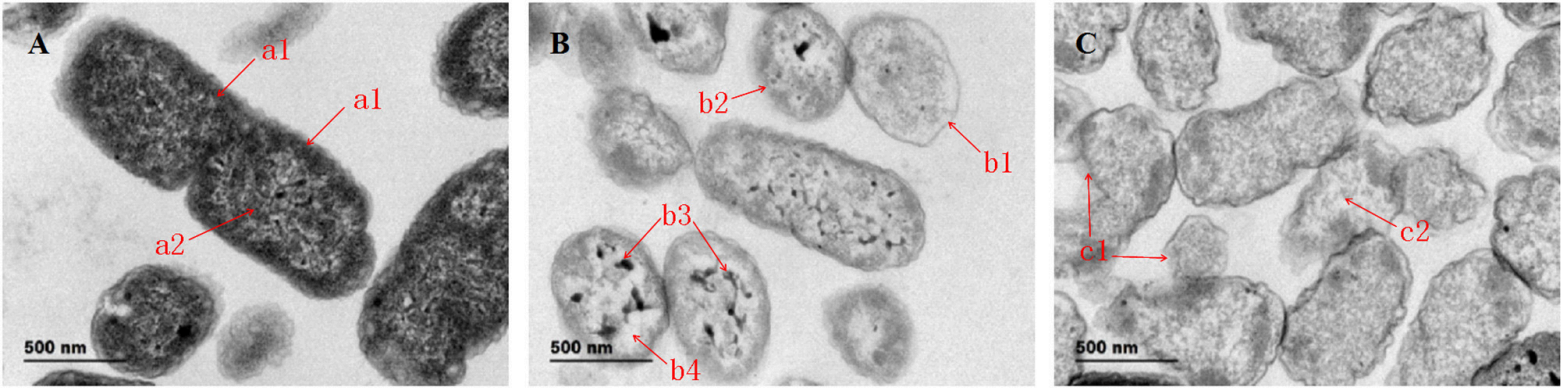
TEM images of *Salmonella* before and after treated with 1,8-cineole, **(A)** Control; **(B)** 1 × MIC for 3 h; **(C)** 1 × MIC for 6 h. Bar = 500 nm. Magnification: × 7000. **(A)** a1 refers to cell wall and membrane with whole structure; a2 refers to cytoplasm without damage. **(B)** b1 refers to cell walls and membranes with thin structure; b2 refers to cell walls and membranes with sectional shrinkage or rupture; b3 refers to the intracellular cytoplasm gathered into a network with nodes; b4 refers to obvious cell cavities. **(C)** c1 refers to the complete rupture cell walls and cell membranes; c2 refers to *Salmonella* sp. D194-2 broken with no cytoplasm left in it.

Figure 1 shows the morphological changes in *Salmonella* sp. D194-2 treated with 1 × MIC CIN for different time periods. In comparison with untreated control cells, surface structure of the treated *Salmonella* sp. D194-2 cells had clearly changed. *Salmonella* sp. D194-2 without CIN treatment (Figure 1A) presented clear, smooth, rod-shaped surface outlines, integral and thick cell walls and cell membranes, and well-distributed intracellular cytoplasm. After treatment for 3 h, structure of the cell walls and membranes thinned and blurred with sectional shrinkage or rupture, while the intracellular cytoplasm gathered into a network with nodes accompanied by obvious cell cavities (Figure 1B). Moreover, there was serious surface shrinkage with a complete rupture cell walls and cell membranes after *Salmonella* sp. D194-2 was treated for 6 h, while some of the cells were broken with no cytoplasm left in them (Figure 1C). Therefore, CIN significantly damaged *Salmonella* sp. D194-2 cell walls and integrity of the membrane structure. It also caused significant leakage of intracellular substances which eventually resulted in complete rupture of cell integrity and cell death. The changes were even more evident with increased in the CIN treatment time.

### Primary data analysis of protein profiles

**Figure 2:**
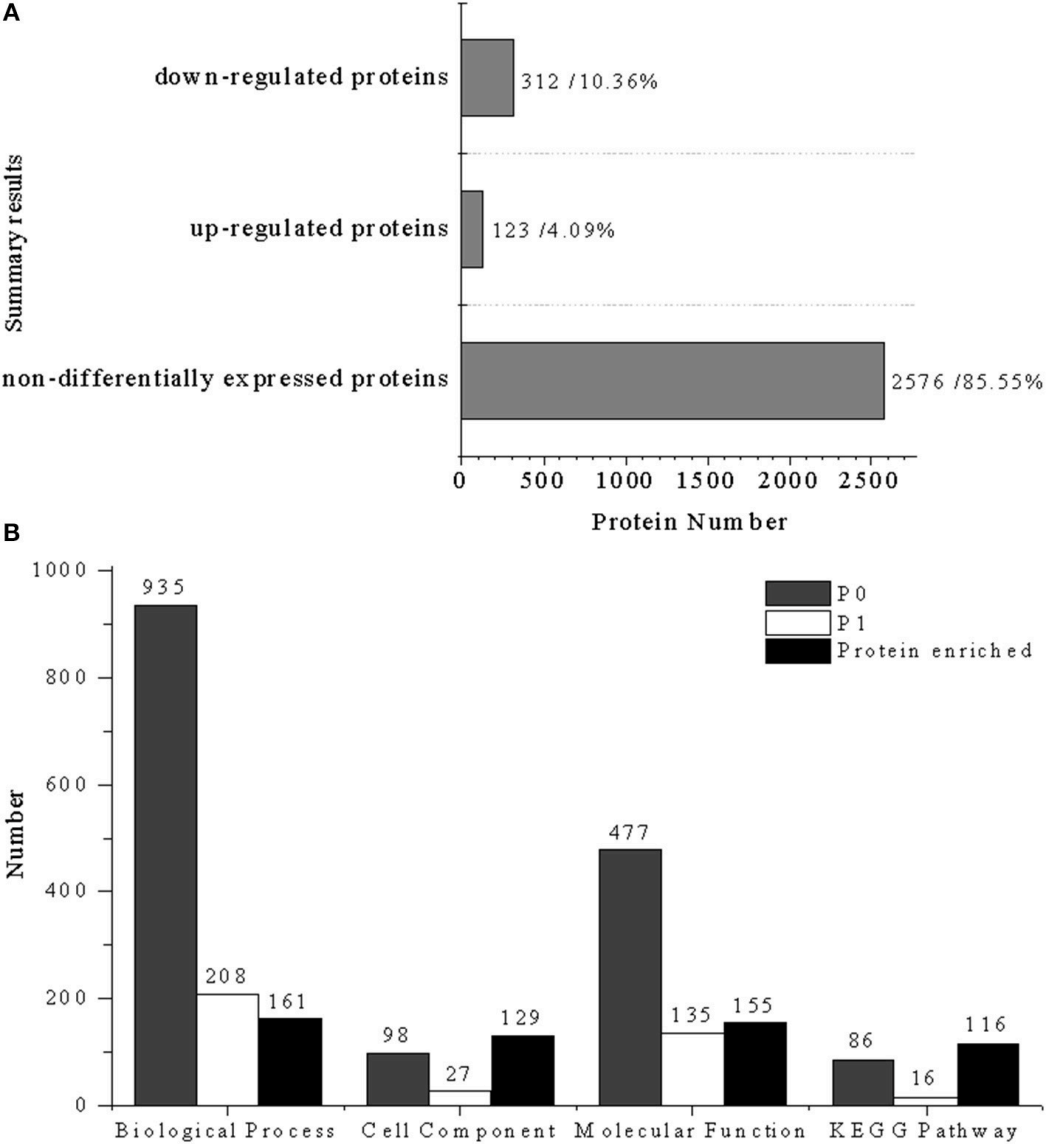
Statistical summary of iTRAQ results: **(A)** Results of differentially expressed proteins: down-regulated proteins (312, 10.36%), up-regulated proteins (123, 4.09%); **(B)** enrichment results of different biological information processes, P0, total enrichment results of biological information processes; P1, significant enrichment results of biological information processes (*P* < 0.05). Biological process (935, 208, 161), cell component (98, 27, 129), molecular function (477, 135, 155), and KEGG pathway (86, 16, 116).

A total of 3011 proteins were detected by iTRAQ analysis from the Uniprot-*Salmonella* database, of which 435 proteins (14.45%) were significantly differentially expressed and 2576 (85.55%) were non-differently expressed proteins. Among the differentially expressed proteins, 123 proteins (4.09%) were up-regulated and 312 (10.36%) were down-regulated (Figure 2A). In comparison to control group, most proteins from the cells treated with CIN clearly decreased, damaging the normal metabolic activity in the bacteria.

**Figure 3:**
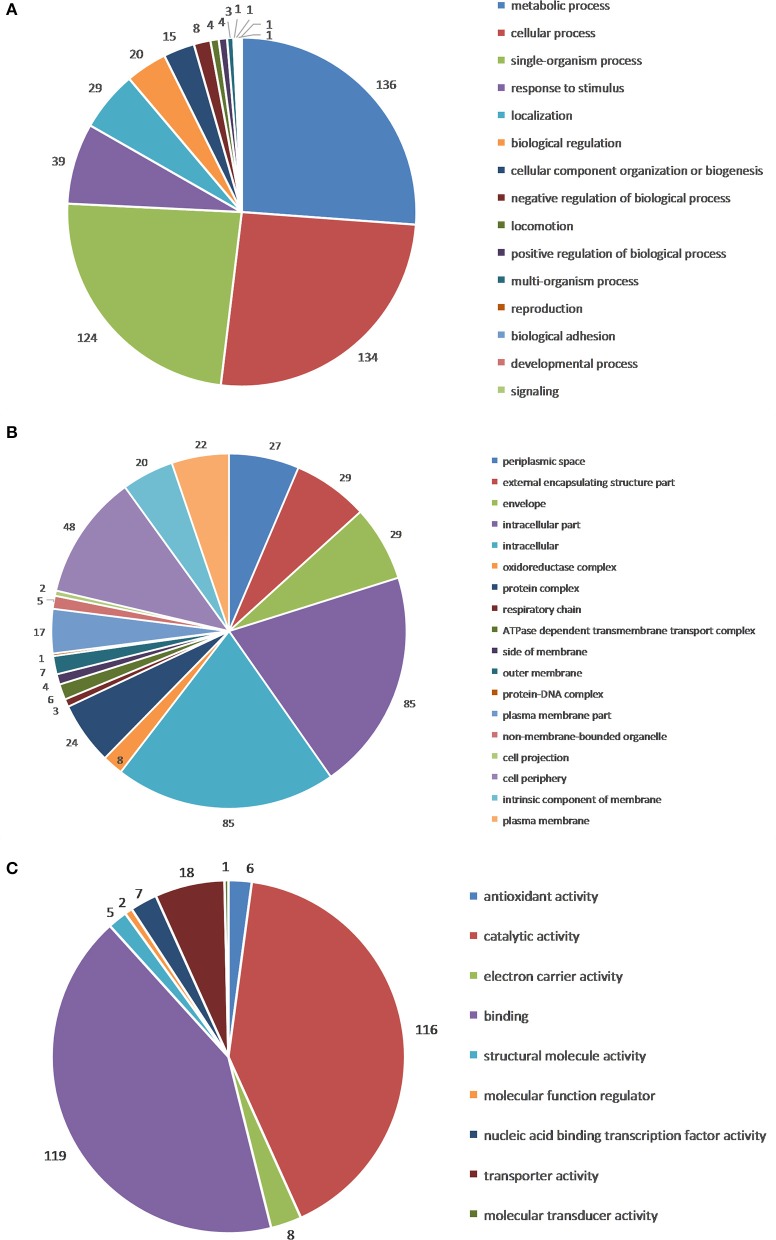
Gene ontology categories of differentially expressed proteins: Biological Process **(A)**, Cellular Component **(B)**, and Molecular Function **(C)**. The differential proteins identified were screened and classified according to online database David 6.7 (http://david.abcc.ncifcrf.gov/) and QuickGO (http://www.ebi.ac.uk/QuickGO/), three GO categories, including Biological Process, Cellular Component, and Molecular Function, were enriched.

**Figure 4:**
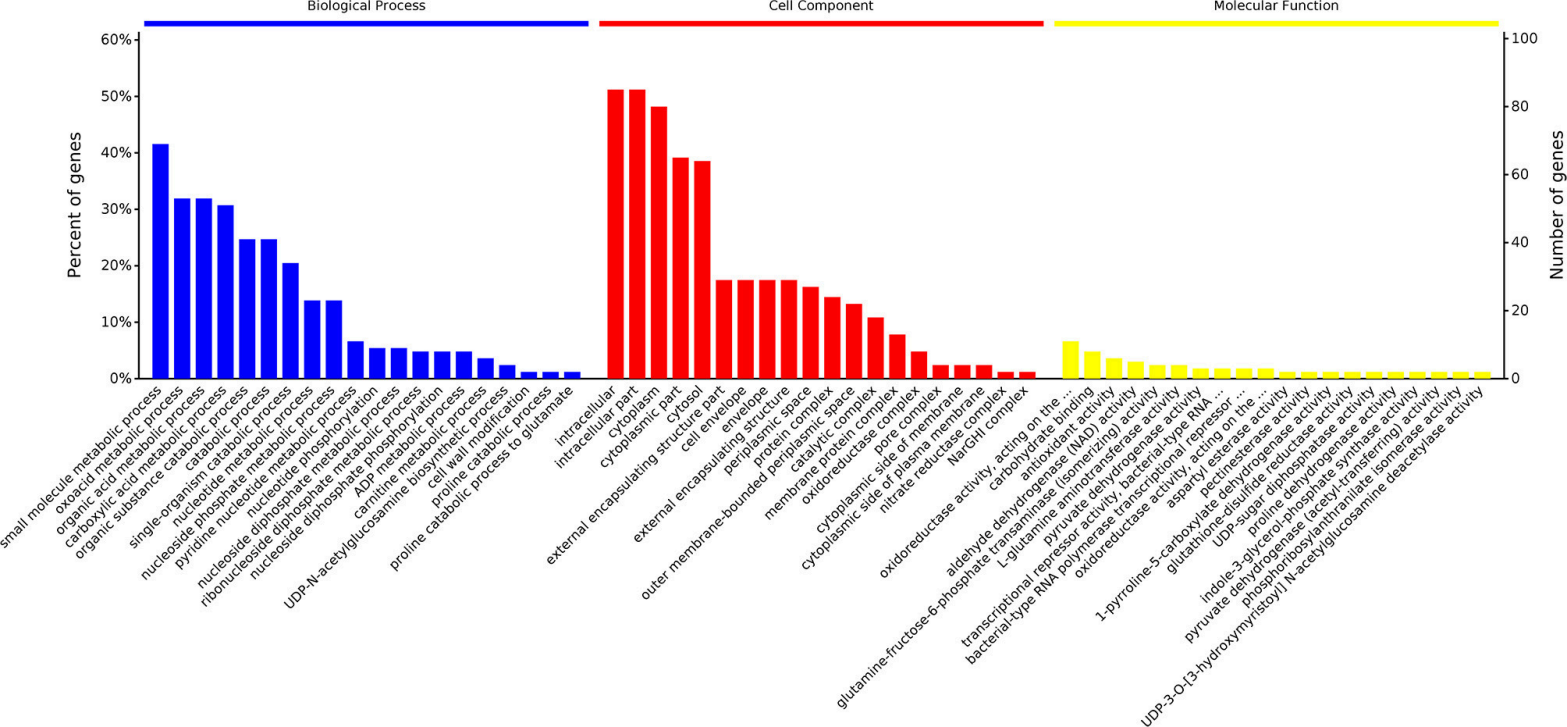
Percent of genes of GO functional annotation results: Biological Process (BP); Cellular Component (CC), and Molecular Function (MF). For each GO ontology, significance of each gene decreases from left to right (*P* < 0.05). The differential proteins identified were screened and classified according to the online database David 6.7 (http://david.abcc.ncifcrf.gov/) and QuickGO (http://www.ebi.ac.uk/QuickGO/), three GO categories, including Biological Process, Cellular Component, and Molecular Function, were enriched. Percent of genes of GO functional annotation results were performed using Microsoft Excel 2007.

GO functional annotation of up-regulated and down-regulated proteins was made in relation to three GO terms: Biological Process (BP), Cellular Component (CC), and Molecular Function (MF). As shown in Figure 2B, 161 up- or down-regulated proteins were enriched in 935 intracellular biological processes of *Salmonella* sp. D194-2; 208 from 935 were in significant response to the CIN treatment (*P* < 0.05). Biological processes of cell wall modification, as well as amino acid, acid, small molecule, katabolic, nucleotide, and phosphate metabolism were clearly stimulated by CIN (Figures 3, 4). The 129 up- or down-regulated proteins were divided into 98 cellular components concentrating on cell walls, cell membranes, and intracellular cytoplasm, such as the periplasmic space, inner and outer membrane, porin, cytosol, oxidoreductase, and catalyzing enzymes. The proteins expressed differently were mainly cytoplasmic components (Figures 3, 4). Furthermore, 155 differential proteins were annotated with 477 molecular functions, while 135 functions were significantly correlated with CIN treatment of *Salmonella* sp. D194-2. CIN may inhibit cell metabolism by decreasing the activity of most of the intracellular catalyzing enzymes (Figures 3, 4).

### Analysis of KEGG pathway

According to the KEGG results (Figure 2B), 116 differential proteins were related to 86 pathways, 16 of which were significant enrichment pathways (*P* < 0.05). These proteins were mainly involved with 29 internal metabolic pathways (Table S3) in *Salmonella* concentrated on global and overview maps, metabolism, genetic information processing, environmental information processing, cellular processes, and human diseases. Proteins connected with carbohydrate metabolism accounted for 62.93% of the annotation proteins, which indicated that carbohydrate metabolism was sensitive to CIN treatment. The top 16 significant pathways are listed in Tables S1, S2 (*P* < 0.05). Pathways related to glycolysis/gluconeogenesis and the citric acid cycle, as well as purine, pyrimidine, pyruvate, glyoxylic acid, two carboxylic acids, amino sugar and nucleoside carbohydrate metabolism were significantly affected by CIN treatment of *Salmonella* sp. D194-2.

### String analysis of differentially expressed proteins

**Figure 5:**
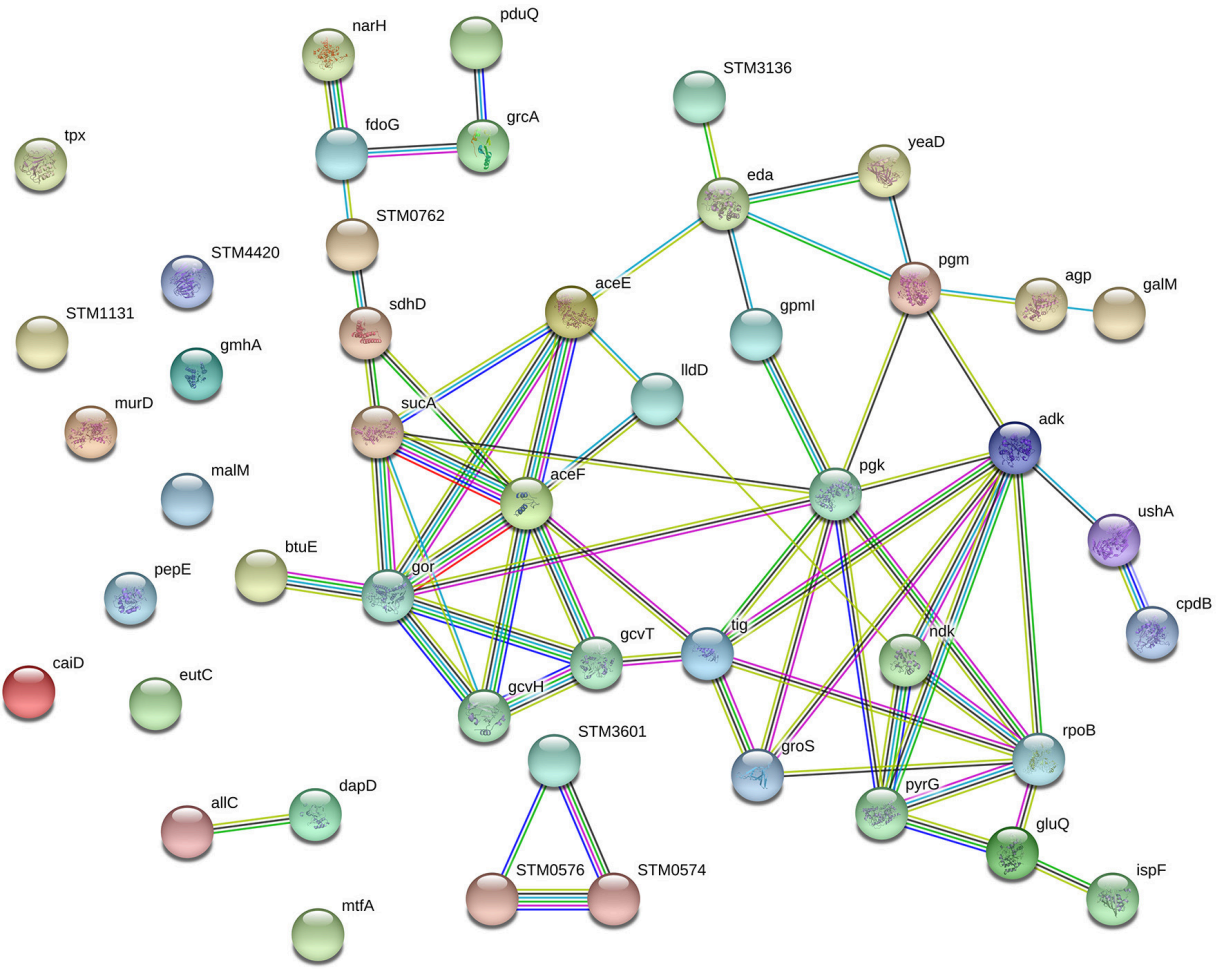
Establishment of protein-protein interaction networks for differential proteins.

The network of proteins was analyzed by String software. Result analysis indicated that most of the differentially expressed proteins were mainly related to carbohydrate metabolism (Figure 5). Protein aceF (Acetyltransferase component of pyruvate dehydrogenase complex, Q8ZRT1), aceE (Pyruvate dehydrogenase E1 component, V1HP36), and pgk (Phosphoglycerate kinase, V1H0S6) take an interrelation in carbohydrate metabolism pathway.

### Validation of proteomics results at mRNA levels

**Figure 6:**
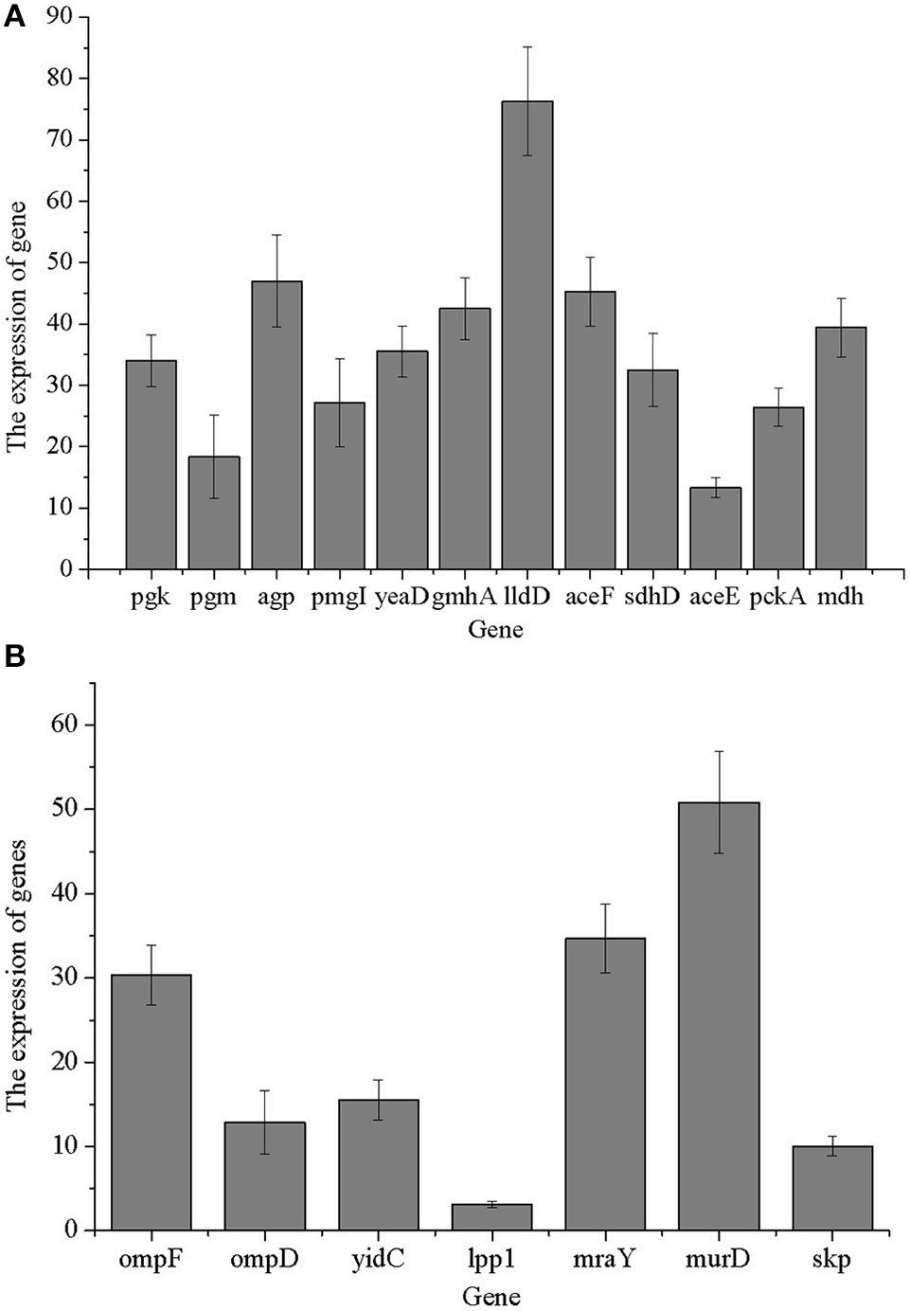
Validation of proteomics results at mRNA levels. **(A)** Genes related to carbohydrate metabolism; **(B)** genes related to cell wall/membrane/envelope biogenesis.

We verified the proteomics results at the mRNA level. Quantification of selected 33 genes was performed. As shown in Figure 6, 12 carbohydrate metabolism genes of selected 33 genes were identified, while 7 cell wall/membrane/envelope genes of selected 33 genes were identified. Data of RT-PCR were obtained in three independent biological experiments and all the primers used in this study are given in Table S4. The results showed that at the transcription level, the levels of translation-related genes were consistent with the data at the proteomics level. Furthermore, some enzymes (pgk, pgm, yeaD, pgmI, and ompF) that participated in carbohydrate metabolism and membrane protein-related genes was down-regulated at the mRNA level when *Salmonella* was treated with 1,8-cineole. In general, most of the transcription levels of selected altered genes were in accordance with our proteomics results. The most interesting finding of this study was most of the differentially expressed proteins are related to the carbohydrate metabolism.

### Effect of CIN on proteins involved in carbohydrate metabolism

**Figure 7:**
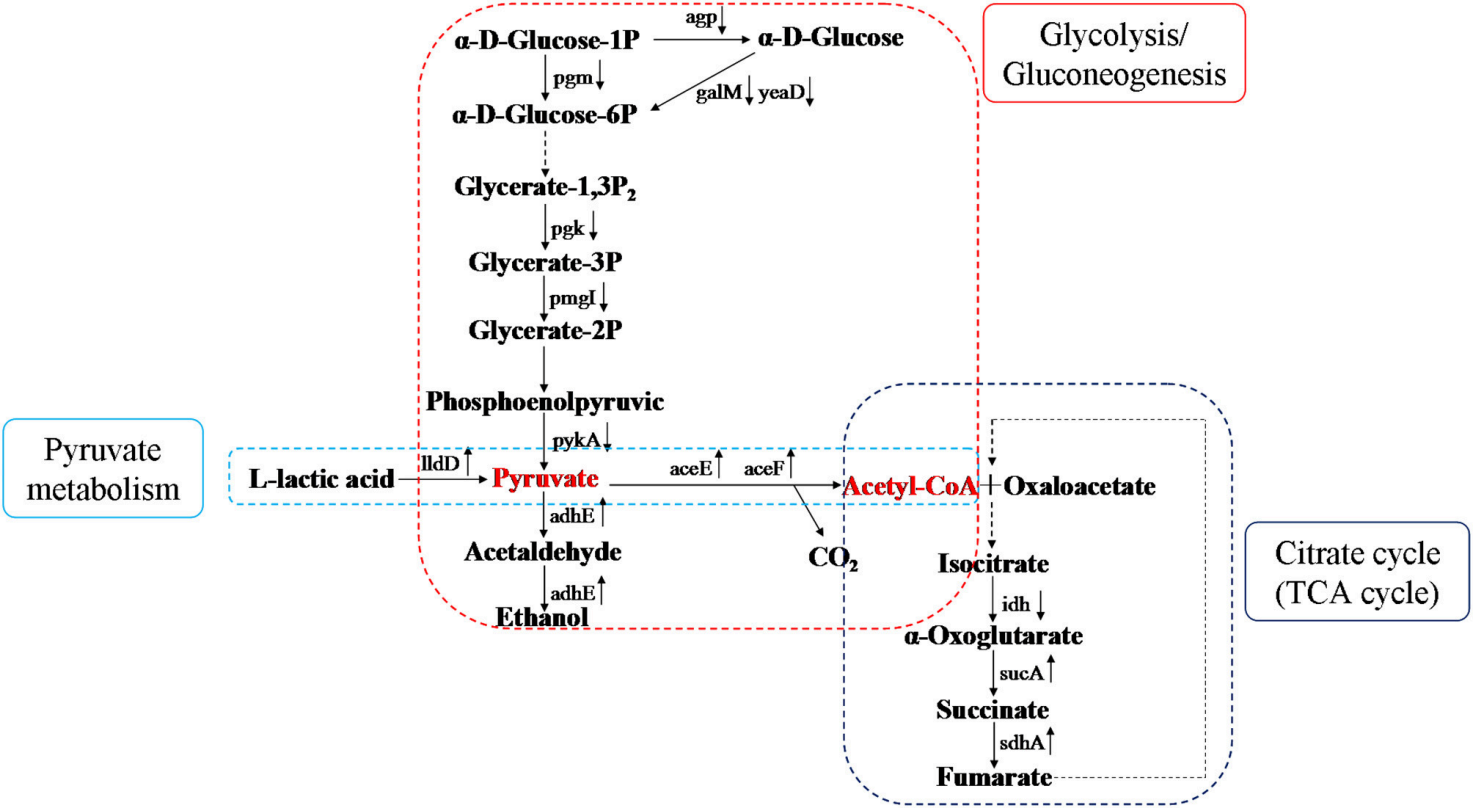
Diagram of differential proteins of carbohydrate metabolism. It indicated changes of differential proteins in three metabolism pathways, and metabolism figure was obtained in the KEGG pathways with up-regulated and down-regulated proteins. Pyruvate is considered as the reference point, pyruvate metabolism is in the left, Glycolysis/Gluconeogenesis is in the middle, and the Citrate cycle is in the right.

According to our previous findings, CIN has a remarkable effect on pathways related to glycolysis/gluconeogenesis, pyruvate metabolism, and citric acid cycle. Glycolysis/gluconeogenesis is a vital metabolic pathway and is connected with many other metabolism pathways through pyruvate or other metabolites. Pyruvate is generated from glucose and metabolized to oxygen and enzymes in bacteria. Under aerobic conditions, acetyl coenzyme A (acetyl-CoA) is generated from pyruvate catalyzed by phosphoenolpyruvate (PEP) carboxylase and is resolved completely through the citric acid cycle. Lactic acid, the deoxidized decomposition product of nicotinamide adenine dinucleotide (NADH) catalyzed by lactate dehydrogenase, is not metabolized completely. Pyruvate and acetyl-CoA are key intermediate products in carbohydrate metabolism. After treatment with CIN, some *Salmonella* enzymes (agp, pgm, galM, yeaD, pgk, pgmI, and pykA) were down-regulated which led to a decrease in pyruvate and an increase in decomposition of pyruvate to acetyl-CoA or ethanol (Figure 7). In significant KEGG Pathway of *Salmonella* (*P* < 0.05), Glycolysis/Gluconeogenesis (eco00010), Pyruvate metabolism (eco00620), and Citrate cycle (TCA cycle) (eco00020) are significant carbohydrate metabolic pathways. In addition, differentially expressed proteins were mapped to KEGG database (http://www.kegg.jp/kegg/pathway.html), the KEGG pathways were enriched, details are all listed in the corresponding table (different protein entries has their corresponding URL links). According to the three metabolism pathways in KEGG database, metabolism figure was obtained in the KEGG pathways with up-regulated and down-regulated proteins. Decomposition of acetyl-CoA was impeded via aerobic metabolism, but promoted via anaerobic metabolism into ethanol.

## Discussion

*Salmonella* is a gram-negative, facultative anaerobic bacterium of the family *Enterobacteriaceae* which is responsible for food poisoning and foodborne disease. Natural anti-bacterial agents have been frequently investigated to find ways to suppress bacteria's resistance. When exposed to biocides, multiple pathways of *Salmonella* involved in adaptation changed and impacted the bacterial-environment interaction (Curiao et al., 2016). From protein levels, post-translational modifications and stability, and protein subcellular localization, proteomics provided an excellent approach to study the biological functions of proteins involved in bacterial-environment interaction.

We screened CIN as a vital EO component and sought to study the antimicrobial effect of CIN on *Salmonella* sp. D194-2. *Salmonella* sp. D194-2 was exposed to 2.5 mg/mL (1×MIC) CIN for 3 and 6 h. Significant damaged to cell envelope was observed by TEM (3 h). Following 6 h of CIN exposure, the cell forms were severely ruptured resulting in cell death. Intolerance to CIN or other EO terpenes may caused the cell membrane disruption of *Salmonella* resulting in uncontrolled loss of cell processes, such as DNA transcription, protein synthesis, and enzyme activity (Gomes-Neto et al., 2014). These results are in accordance with TEM observations of Guerra-Rosas et al. (2017). TEM was used to observe the alterations caused in the structure of *E. coli* and *L. innocua* cells by the action of EO-loaded nanoemulsions. In their work, changes in the cellular structure of both microorganisms after contact with nanoemulsions containing different EOs can be observed in contrast with control cells. Results also showed that CIN exhibited antimicrobial activity against *Salmonella* Enteritidis and *Salmonella* Typhi (Donato et al., 2015). Due to the lipophilic nature of CIN, it is believed that CIN displayed antibacterial activity against Gram-negative pathogenic bacteria by inducing cell membrane disruption (Ojeda-Sana et al., 2013). In their study, the bactericidal effect associated with injury to the cell membrane exerted by CIN on *E. coli* is reported. Following exposure to 2.5 mg/mL (1×MIC) CIN for 3 h, *Salmonella* sp. D194-2 cell morphology was significantly disrupted in nutrient broth. Three hours was used as CIN exposure time to *Salmonella* sp. D194-2 to CIN for further proteomic analysis.

In order to evaluate the effects of CIN exposure, whole cell protein patterns of experimental groups (2.5 mg/mL CIN) were compared to the control groups (0 mg/mL CIN) using iTRAQ. Global analysis showed that 435 out of 3,011 detected proteins were differentially expressed with more than a 1.5-fold up- or down-regulation. More than 85% of the differentially expressed proteins were involved in binding, catalytic, transporter, electron carrier, and antioxidant activity. Further analysis showed that most of the down-regulated proteins were associated with carbohydrate, nucleotide, amino acid, lipid, and energy metabolism in the KEGG pathways.

During carbohydrate metabolism, phosphoglucomutase (pgm) catalyzes the interconversion between glucose-1-phosphate and glucose-6-phosphate and maintains them at dynamic balance, so pgm is pivotal in glucose metabolism. Glucose- 1-phosphate is an intermediate in the glycolysis of glucose and lactose metabolism, and also the precursor of biosynthetic sugar nucleotide and the component of lipopolysaccharide core in the center of lipopolysaccharides. Cell walls of gram-negative bacteria are composed of peptidoglycan, lipopolysaccharides, lipoproteins, and teichoic acid. Thus, the differential expressions of pgm not only cause the significant enrichment of multiple metabolic pathways, but also largely affect cell structure of *Salmonella*.

Our results demonstrated significant changes in carbohydrate metabolism upon administration with CIN. A variety of proteins related to metabolism were differentially expressed after treatment with CIN, including up-regulation of proteins from L-lactate dehydrogenase, acetyltransferase component of pyruvate, succinate dehydrogenase hydrophobic membrane anchor subunit, acetyltransferase component of pyruvate dehydrogenase complex, and downregulation of proteins from isocitrate dehydrogenase, phosphoglycerate kinase, phosphoglucomutase, 2,3-bisphosphoglycerate-independent phosphoglycerate mutase, and glucose-1-phosphatase. Acetyl-CoA produced from pyruvate can be used to generate ATP from ADP by conversion to acetate or to maintain redox balance by conversion to ethanol. It is generally accepted that aldehyde-alcohol dehydrogenase (adhE) perform the important function of regenerating NAD^+^ from NADH to maintain a continuous flow of glycolysis (Atteia et al., 2013; Pineda et al., 2013). Aldehyde-alcohol dehydrogenase (adhE, D7VE84) was significantly upregulated in this study, perhaps promoting the production of ATP and NAD^+^ during pyruvate metabolism when *Salmonella* sp. D194-2 was exposed to CIN (Wu et al., 2016).

A recent transcriptome analysis revealed that protein acetylation is involved in the virulence of S. Typhimurium by having an effect on invasion, intracellular survival and systemic infection (Sang et al., 2016). The relative activities of key enzymes involved in glycolysis and citrate cycle pathway are all regulated by Pat and CobB. Central metabolism plays an important role in the infection of a host by a pathogen. Studies have proposed that acetylation modification occurs in Phosphate Fructose Kinase A (PfkA) and that the metabolism alteration mediated by acetylation may partially contribute to S. Typhimurium's virulence (Liu et al., 2014; Sang et al., 2016).

Lipid A is an important component of the outer membrane of gram-negative bacteria. As an essential enzyme in the biosynthesis of the lipid A component, LpxC inhibits the conversion of UDP-N-acetylglucosamine into acyl-ACP, thereby promoting the accumulation of acyl-ACP (McClerren et al., 2005; Barb and Zhou, 2008). Experiments have highlighted the potential of LpxC inhibitors as a new class of antibiotic against fatal infections caused by extremely virulent pathogens (Lemaître et al., 2017). Most of small-molecule LpxC inhibitors possess a Zn^2+^-binding hydroxamate moiety as well as a structural element addressing the hydrophobic tunnel of the enzyme. Due to the lipophilic nature of 1, 8-cineole (CIN), it is believed that the lipophilic side chain of CIN penetrates through the hydrophobic tunnel of LpxC, their hydroxamate moiety chelates the catalytic Zn^2+^-ion in the active site. Thus, CIN displayed antibacterial activity against Gram-negative pathogenic bacteria (Ojeda-Sana et al., 2013; Tangherlini et al., 2016). Our results demonstrate that CIN down-regulated LpxC 0.63-fold and may act as a LpxC inhibitor in *Salmonella* sp. D194-2. As natural antibacterial agents, CIN cannot encourage S. Typhimurium to develop direct tolerance or cross-tolerance (Gomes-Neto et al., 2014). Therefore, CIN may have potential for a role in the clinical development of new antibiotics to combat *Salmonella* infections.

## Conclusions

In summary, this study shows significant change in the carbohydrate, nucleotide, amino acid, lipid and energy metabolism of *Salmonella* sp. D194-2 under CIN stress. The up-regulated adhE may promote production of ATP and NAD^+^ in *Salmonella* sp. D194-2 when exposed to CIN. We showed that carbohydrate metabolism and membrane protein-related genes was down-regulated at the mRNA level when *Salmonella* was treated with 1,8-cineole. Therefore, CIN has the potential to play a key role in the clinical development of new antibiotics for treatment of *Salmonella* infections.

## Author contributions

XC contributed equally to this work, including experiment and the writing of manuscript. YS wrote and edited the manuscript. JC provided some help to the experiment. ZW participated in the revision of manuscript. DP participated in the writing of manuscripts.

### Conflict of interest statement

The authors declare that the research was conducted in the absence of any commercial or financial relationships that could be construed as a potential conflict of interest.
