# Supplementary material for: Effects of 1,8-cineole on Carbohydrate Metabolism Related Cell Structure Changes of Salmonella
Source: Front Microbiol. 2018 May 25;9:1078. doi: 10.3389/fmicb.2018.01078 (PMC5992416; doi:10.3389/fmicb.2018.01078)
Supplement: Supplementary file 1 [file Data_Sheet_1.docx]

Supplementary data

Table. 1 Significant KEGG Pathway of *Salmonella* differentially expressed proteins (*P <* 0.05).

| Pathway Name | ID | P value | Count | Ratio  (%) |
| --- | --- | --- | --- | --- |
| Insulin resistance | eco04931 | 0 | 4 | 3.45 |
| Biosynthesis of antibiotics | eco01130 | 3.15E-07 | 35 | 30.17 |
| Glycolysis / Gluconeogenesis | eco00010 | 3.74E-06 | 13 | 11.21 |
| Microbial metabolism in diverse environments | eco01120 | 0.0000552 | 34 | 29.31 |
| Biosynthesis of secondary metabolites | eco01110 | 0.00017 | 38 | 32.76 |
| Carbon metabolism | eco01200 | 0.000519 | 18 | 15.52 |
| Citrate cycle (TCA cycle) | eco00020 | 0.00244 | 7 | 6.03 |
| Metabolic pathways | eco01100 | 0.00692 | 65 | 56.03 |
| Glyoxylate and dicarboxylate metabolism | eco00630 | 0.008 | 8 | 6.9 |
| Pyruvate metabolism | eco00620 | 0.0098 | 9 | 7.76 |
| Amino sugar and nucleotide sugar metabolism | eco00520 | 0.016 | 8 | 6.9 |
| Purine metabolism | eco00230 | 0.0213 | 12 | 10.34 |
| Alanine, aspartate and glutamate metabolism | eco00250 | 0.0219 | 6 | 5.17 |
| Pyrimidine metabolism | eco00240 | 0.0396 | 9 | 7.76 |
| Glutathione metabolism | eco00480 | 0.0448 | 4 | 3.45 |
| Geraniol degradation | eco00281 | 0.0459 | 2 | 1.72 |

ID means the Identification number of the Pathways. Count represents the number of total differential proteins enriched in the biological process, while the Ratio represents the ratio of the number of differential proteins involved in the pathway to that of total differential proteins of all KEGG pathways.

Table. 2 Enrichment of differential expressed proteins of 16 significant KEGG pathways (*P*< 0.05)

| ID | Fold Change | Protein Name | Gene |
| --- | --- | --- | --- |
| Q8ZKS8 | 1.6952987 | Formate dehydrogenase | fdoG |
| Q8ZQU1 | 1.6026101 | 2-oxoglutarate dehydrogenase decarboxylase component | sucA |
| Q8ZRT1 | 1.9776031 | Acetyltransferase component of pyruvate dehydrogenase complex | aceF |
| S4KRJ5 | 0.452991 | Isocitrate dehydrogenase [NADP] | A678_00591 |
| G5QEL5 | 0.6234522 | Trigger factor | tig |
| V1H0S6 | 0.4070672 | Phosphoglycerate kinase | pgk |
| Q8ZQW9 | 0.661827 | Phosphoglucomutase | pgm |
| G5QRI3 | 1.995915 | L-lactate dehydrogenase | lldD |
| B3YKT3 | 0.3341401 | Aminomethyltransferase | gcvT |
| S4K7L7 | 0.4625043 | Aldehyde dehydrogenase B | A678_03133 |
| B5R6G8 | 0.5658897 | Glucose-1-phosphatase (G1Pase), secreted | agp |
| Q7CPT2 | 0.6433833 | Putative D-mannonate oxidoreductase | STM3136 |
| B5RGH4 | 0.6602949 | 2,3-bisphosphoglycerate-independent phosphoglycerate mutase | pmgI |
| A0A0T9VKC4 | 0.298764 | Carnitinyl-CoA dehydratase | caiD |
| V1HAP2 | 0.3989925 | Autonomous glycyl radical cofactor | grcA |
| Q8ZK62 | 0.659048 | Putative inner membrane protein | STM4420 |
| S4K4G6 | 0.6346003 | Mannose-6-phosphate isomerase | A678_01678 |
| S4KAB8 | 0.4437362 | Probable thiol peroxidase | tpx |
| Q8ZNW4 | 0.4840116 | 2-keto-3-deoxygluconate 6-phosphate aldolase | eda |
| A0A0U1GWP5 | 0.5980426 | Aldose 1-epimerase | yeaD |
| Q8ZLD0 | 0.6690788 | Putative phosphosugar isomerase | STM3601 |
| Q8ZQS1 | 0.5414908 | Aldose 1-epimerase | galM |
| A9MNV5 | 0.6219622 | Uncharacterized protein | SARI_04254 |
| A0A0W4M6J8 | 0.6543855 | Peptidase E | pepE |
| A0A0W4RBE9 | 0.6478299 | Transaldolase | tal |
| Q9XDN0 | 1.6444353 | Propanediol utilization propanol dehydrogenase | pduQ/adhE |
| A0A0W4BHJ7 | 0.6593326 | Ethanolamine utilization protein EutG | A6C39_19730 |
| S4KG00 | 0.5273033 | 10 kDa chaperonin | groS |
| Q8ZR46 | 0.3937015 | Putative transport protein, PTS system | STM0576 |
| A0A0W3R3E0  Q8ZQT0 | 0.2939636  0.5923369 | Glycine cleavage system H protein  Fumarate hydratase, alpha subunit | gcvH  STM0762 |
| A0A0V2F0K6 | 0.5599759 | DNA partition complex ParG | LFZ31_22775 |
| B5R684 | 1.6087651 | Succinate dehydrogenase hydrophobic membrane anchor subunit | sdhD |
| Q8ZR48 | 0.5920935 | Putative transport protein, PTS system | STM0574 |
| V1HP36 | 1.5471199 | Pyruvate dehydrogenase E1 component | aceE |
| S4KH74 | 0.6411936 | Uncharacterized protein | A678_02101 |
| A0A0W3QE96 | 1.5657456 | DNA-directed RNA polymerase subunit beta | rpoB |
| S4K9T9 | 1.5520702 | CTP synthase | pyrG |
| A0A0T9VE95 | 0.4830381 | 2',3'-cyclic-nucleotide 2'-phosphodiesterase | cpdB |
| A9MLY5 | 0.3716492 | Adenylate kinase | adk |
| S4JT09 | 0.6655074 | Thioredoxin reductase | A678_04909 |
| Q8ZR77 | 0.6230064 | Allantoate amidohydrolase | allC |
| B3YFJ9 | 0.5530469 | Nucleoside diphosphate kinase | ndk |
| Q8ZQ33 | 1.5583323 | Putative outer membrane protein | STM1131 |
| S4KH87 | 0.6092337 | Protein MtfA | mtfA |
| V1GL64 | 0.6000678 | DNA polymerase III subunit theta | SEI61121_20856 |
| P06196 | 0.6366169 | Silent protein UshA(0) | ushA |
| A0A0W4MGL3 | 1.669499 | Bifunctional protein PutA | putA |
| A0A0W3UGV1 | 0.6659714 | Methylmalonate-semialdehyde dehydrogenase | mmsA |
| V1H328 | 0.5890332 | 2,3,4,5-tetrahydropyridine-2,6-dicarboxylate N-succinyltransferase | dapD |
| V1H0V8 | 1.6357393 | L-threonine dehydratase catabolic TdcB | SEI61121_13804 |
| G5QQR1 | 0.4778738 | OsmC/Ohr family protein | LTSERUB_5443 |
| V1H2X8 | 0.5478992 | Superoxide dismutase [Cu-Zn] | SEI61121_04536 |
| Q8ZLD4 | 0.6589249 | Glutathione oxidoreductase | gor |
| S4K2J3 | 0.6141857 | UDP-N-acetylmuramoylalanine-D-glutamate ligase | murD |
| A0A0W4TTE2 | 0.3741826 | Ethanolamine ammonia-lyase light chain | eutC |
| A0A0U1F6R2 | 0.5208717 | Maltose operon periplasmic protein | malM |
| Q7CQN7 | 0.5611541 | Thioredoxin/glutathione peroxidase BtuE | btuE |
| A9MIB9 | 0.4036325 | Uncharacterized protein | SARI_00420 |
| V1GT39  A0A0T9VN06 | 1.7993505  0.668694 | Nitrate reductase A subunit beta  NH(3)-dependent NAD(+) synthetase | narH  nadE_1 |
| G5LRY5 | 0.6239343 | Uncharacterized protein | LTSEALA_3708 |
| V1GVT3 | 1.8280638 | Glutamyl-Q tRNA(Asp) synthetase | gluQ |
| B3YJ79 | 0.5999621 | 2-C-methyl-D-erythritol 2,4-cyclodiphosphate synthase | ispF |
| S4LGS6 | 0.4148819 | Phosphoheptose isomerase | gmhA |
| S4KFM5 | 0.6348277 | Uncharacterized protein | A678_02528 |
| S4JKT1 | 0.5090937 | Oxidoreductase, FAD/FMN-binding protein | A678_04495 |
| S4K4Y0 | 0.5873418 | Oxygen-insensitive NAD(P)H nitroreductase | A678_02878 |
| S4KCR5 | 0.6138782 | Class B acid phosphatase | A678_02144 |
| S4JX41 | 0.5581686 | Uncharacterized protein | A678_02960 |

ID means the Identification number of the Pathways.

Table. 3 KEGG Pathway category of differentially expressed proteins

| Pathway Name | Count | Ratio/% |
| --- | --- | --- |
| **Global and overview maps** | | |
| Biosynthesis of antibiotics | 35 | 30.17 |
| Microbial metabolism in diverse environments | 34 | 29.31 |
| Biosynthesis of secondary metabolites | 38 | 32.76 |
| Carbon metabolism | 18 | 15.52 |
| Metabolic pathways | 65 | 56.03 |
| Fatty acid metabolism | 3 | 2.59 |
| Biosynthesis of amino acids | 11 | 9.48 |
| Degradation of aromatic compounds | 1 | 0.86 |
| 2-Oxocarboxylic acid metabolism | 1 | 0.86 |
| **Metabolism** | | |
| Carbohydrate metabolism | 73 | 62.93 |
| Energy metabolism | 8 | 6.90 |
| Lipid metabolism | 8 | 6.90 |
| Nucleotide metabolism | 21 | 18.10 |
| Amino acid metabolism | 29 | 25.00 |
| Metabolism of other amino acids | 9 | 7.76 |
| Glycan biosynthesis and metabolism | 4 | 3.45 |
| Metabolism of cofactors and vitamins | 10 | 8.62 |
| Metabolism of terpenoids and polyketides | 4 | 3.45 |
| Biosynthesis of other secondary metabolites | 1 | 0.86 |
| Xenobiotics biodegradation and metabolism | 9 | 7.76 |
| **Genetic Information Processing** | | |
| Transcription | 1 | 0.86 |
| Translation | 5 | 4.31 |
| Folding, sorting and degradation | 5 | 4.31 |
| Replication and repair | 5 | 4.31 |
| **Environmental Information Processing** | | |
| Membrane transport | 18 | 15.52 |
| Signal transduction | 9 | 7.76 |
| **Cellular Processes** | | |
| Cell motility | 3 | 2.59 |
| **Human Diseases** | | |
| Endocrine and metabolic diseases | 4 | 3.45 |
| Drug resistance: Antimicrobial | 3 | 2.59 |

Count represents the number of total differential proteins enriched in the KEGG pathway, while the Ratio represents the ratio of the number of differential proteins involved in the pathway to that of total differential proteins of all KEGG pathways.

Table. 4 Primers used for RT-PCR

| **genes** | **primer (5'-3')** |
| --- | --- |
| 16s RNA1-F | ACCCTTATCCTTTGTTGCC |
| 16s RNA1-R | ATGAGGTCCGCTTGCTCT |
| 16s RNA2-F | CCTTTGTTGCCAGCGATTA |
| 16s RNA2-R | CGGACTACGACGCACTTTATG |
| A678_00591-F | CTTCCCGCTGAAACCCTT |
| A678_00591-R | CTTGGCGTGCCCTGATAG |
| A678_02693-F | TTCCGCATTCATACCTTT |
| A678_02693-R | GCAATATCTTTCCCTTCCT |
| aceE-F | CTGGTGGCTGACTGGACT |
| aceE-R | TGGTATGGGCGAGGATTA |
| aceF-F | TACACGCCAGTGGTCTTCA |
| aceF-R | CTCGGTCACGCTCTTCTTAT |
| agp-F | GCTGATACCGTCGGGAGA |
| agp-R | AGGTAGGGTCCATAGTGCC |
| atpD-F | ATGAACGTCCTGGGCGAACC |
| atpD-R | ACCCGCACCACCGAACAGAC |
| caiA-F | AGCGGATAACGGCACCAT |
| caiA-R | GCATTTCATCAGAACCACCA |
| caiB-F | GTATGACGAAAGGGAAAGACC |
| caiB-R | CGGCGTACCGAGAATGTG |
| cybC-F | CTGGTATTCGGTTCGGCTGTT |
| cybC-R | GGGCTATCCGGCGCTTTA |
| fabD-F | CTGGATGATGCCTCTATTGC |
| fabD-R | GCGTTCTACCGCCTCTTT |
| fadL-F | TTTTGCGGGCGATTTAGG |
| fadL-R | CTTCCGAGCGGTAGGTCA |
| ghrB-F | GGCAGGTCTGGATGTCTTTG |
| ghrB-R | CGGATTGACGCAGTTCTTCTC |
| GlpF-F | GGCTATTTGCCTGTTTCG |
| GlpF-R | TGAGGATTCGGGTAAGTTGA |
| gmhA-F | AAGCGGGTGGTAAGGTAC |
| gmhA-R | GAGATATGGCTAACATCAGAAA |
| lldD-F | TGCTCCACGCTGGTCTTT |
| lldD-R | ACATCCCACGCCCATTTA |
| lpp1-F | GCGGTAATCCTGGGTTCT |
| lpp1-R | ATTGCGTTCACGTCGTTG |
| lsrB-F | GAACCAGTGGGTGAAAGAA |
| lsrB-R | TAAAGCGTTAGCGTCAGG |
| mdh-F | GCCGAGCTGACTAAACGT |
| mdh-R | TACTGACCATCGCCTTCC |
| mraY-F | GAATCGCACTTCAGTAAACG |
| mraY-R | GTATCTTTCCGCACCACTTT |
| murD-F | CGCCCAACAGACTGAGACG |
| murD-R | CCAGACGGGTAAAGACATCG |
| ompD-F | CAGCGAGCAACCTGAATG |
| ompD-R | GTCGGACTGATACTGAGCAACT |
| ompF-F | TGCCGACCAGACTTATGC |
| ompF-R | AGCCAGACGGACCAGATT |
| pckA-F | AAGGGATGTTCTCGGTAATG |
| pckA-R | TCGGTGGACAGAGTGGTT |
| pgk-F | TCGTCCGATGGTGGCTAT |
| pgk-R | GCTTCATACAGGGATTTACCG |
| pgm-F | ACTGGAAACGTATCGCTGAG |
| pgm-R | ATCGCACATTCGGAGGAG |
| pmgI-F | ACAGCCACGAAGATCACATT |
| pmgI-R | ACCGACGATAGACGCCAC |
| sdhD-F | CATCCTGGTCCGTGCTAC |
| sdhD-R | GCAGGGTGAAGACTTTGGT |
| skp-F | GCAGAAGACGGGTGTATC |
| skp-R | CTACCTGCTTTCATGGATT |
| UcpA-F | TTCCGCTACGCCGTCTTG |
| UcpA-R | CGCCTACGCTTACGCTTT |
| YeaD-F | GCGAGGAAGAGGTATTGTG |
| YeaD-R | ACGGCAGACCCTGTTGAG |
| yidC-F | CATCGCTTACCTGAAATCC |
| yidC-R | TTGGTGAAGTCGCTGTCG |
